# Expression of Concern: Exosomes function as nanoparticles to transfer miR-199a-3p to reverse chemoresistance to cisplatin in hepatocellular carcinoma

**DOI:** 10.1042/BSR-2019-4026_EOC

**Published:** 2022-06-14

**Keywords:** DDP, Exosome, hepatocellular carcinoma, Huh-7/DDP, miR-199a-3p

The Editorial Office has been made aware of potential issues surrounding the scientific validity of this paper, including identical image panels that appear across multiple publications. Figure panels 3C and 3D contains identical images with the following papers: ‘The Long Non-coding RNA LINC01705 Regulates the Development of Breast Cancer by Sponging miR-186-5p to Mediate TPR Expression as a Competitive Endogenous RNA’ (Figure panels 7C and 7D respectively; https://doi.org/10.3389/fgene.2020.00779), ‘Circular RNA circ-0016068 Promotes the Growth, Migration, and Invasion of Prostate Cancer Cells by Regulating the miR-330-3p/BMI-1 Axis as a Competing Endogenous RNA’ (Figure 2C and 2D respectively; https://doi.org/10.3389/fcell.2020.00827), and ‘LncRNA SNHG4 promotes osteosarcoma proliferation and migration by sponging miR‐377‐3p’ (Figure 2D; https://doi.org/10.1002%2Fmgg3.1349). Figure panels 3E and 3F share identical images with those of article ‘Long Non-Coding RNA ELFN1-AS1 Promoted Colon Cancer Cell Growth and Migration via the miR-191-5p/Special AT-Rich Sequence-Binding Protein 1 Axis’ Figure panel 3C (https://doi.org/10.3389%2Ffonc.2020.588360). The authors have been contacted with regards to these concerns.

